# Achieving Systemic and Scalable Private Sector Engagement in Tuberculosis Care and Prevention in Asia

**DOI:** 10.1371/journal.pmed.1001842

**Published:** 2015-06-23

**Authors:** William A. Wells, Mukund Uplekar, Madhukar Pai

**Affiliations:** 1 US Agency for International Development, Washington, D.C., United States of America; 2 Global TB Programme, World Health Organization, Geneva, Switzerland; 3 McGill Global Health Programs and McGill International TB Centre, McGill University, Montreal, Quebec, Canada

## Abstract

William Wells and colleagues describe opportunities for improving public-private health provider partnerships to tackle TB.

**Summary Points**

- Tuberculosis (TB) is a major public health threat. But worldwide, the majority of people with symptoms consistent with TB start their care seeking in the private or informal sector. These numbers are particularly high in Asia.
- Public-private mix (PPM) efforts have been introduced to reach these individuals, as soon as possible, with quality-assured diagnosis and treatment. Systematic approaches have been designed to reach all provider types. However, PPM schemes struggle to manage the scale of a fragmented and under-regulated private sector.
- Opportunities are arising to introduce more systemic, scalable, and innovative approaches, including social businesses, insurance-based initiatives, intermediary agencies, regulatory regimes, and provider consolidation, with a heavy emphasis on the use of new information technologies.
- These approaches combine the previous work on TB private sector engagement with structural solutions that make health systems function for all patients, regardless of the disease or whether patients seek care in the public or the private sector.

## Health-Seeking Behaviors

As a transmissible, airborne disease, tuberculosis (TB) is a classic public health issue, and the majority of TB prevention and care efforts globally have focused on the public-sector role. However, in sub-Saharan Africa and South Asia, respectively, 49% and 81% of all patients present initially to private or informal (nonqualified) providers [1]. Many of those patients have TB symptoms and a subset have TB disease, but studies show substantial diagnostic delays [2] and high patient costs [3] that are associated with seeing multiple private providers.

Shortening the pathway for those individuals—from their initial private-sector consultation to quality-assured, evidence-based treatment in either the public or private sector—is no simple task. The initial symptoms of TB are nonspecific, diagnosis requires clinical judgment and laboratory testing, and treatment requires long-term monitoring. Nevertheless, tackling this private-sector issue is essential to lessen the TB burden on both patients and, via reduced transmission, communities [4].

TB is one of the first health arenas in which this issue of private-provider involvement in long-term care is being tackled seriously in low-income countries. At the global level, WHO’s new End TB Strategy emphasizes the need for such “bold policies and supportive systems” [5,6]. Here, we outline the current and newer approaches being taken—and the opportunities for more systemic and scalable efforts to engaging private-sector providers in TB control and beyond. Our emphasis is on Asia (based on the large private sector and evidence base), but we expect many conclusions to be broadly applicable.

People do not come to health services labeled as TB patients but rather present with variable and sometimes mild symptoms. For such an individual, a rapid visit to a pharmacy or informal provider, with a (putative) solution dispensed on the spot without a consult fee or prescription, is often far more attractive than a protracted visit and diagnostic process at a public health facility.

In addition to health-seeking evidence from prevalence surveys (Box 1), multiple studies demonstrate a familiar, three-step pattern: an initial consultation with a pharmacist (often with over-the-counter sales of cough medicines and broad-spectrum antibiotics) [7], followed by one or more visits to private providers, and finally, after much delay, TB diagnosis and treatment in the public sector [8]. In India, TB patients interviewed in the public sector had taken particularly circuitous routes to public-sector TB treatment. All [7] or 86% [9] had visited private providers first, and the sequential visits to an average of three [2] formal and informal providers had resulted in substantial delays and costs before the patients eventually made it to the public TB program [7].

**Box 1. Prevalence Surveys Tell the Story**

One side benefit of TB prevalence surveys is a better understanding of health-seeking behaviors related to TB. In Cambodia, for example, the 2011 prevalence survey identified 103 smear-positive individuals, but only four were on TB treatment [10]. Others had no cough (13), had not sought attention (13), or had self-medicated (2). However, that left 71 who were not previously diagnosed or put on treatment despite seeking care for their symptoms at government hospitals (8), health centers (31), private clinics (6), private hospitals (2), and pharmacies (24). All of these represent missed opportunities—in both the public and private sectors—to identify and diagnose TB.

Of course, not all TB patients do get to the public sector for treatment. In 2008–2009, enough TB drugs were sold in the private sector in four countries—India, Pakistan, the Philippines, and Indonesia—to treat potentially 65%–117% of those countries’ TB burdens with a full regimen [11]. It is well documented that such treatment is often of a poor quality [9,12], with use of nonrecommended diagnostics [13], variable TB drug regimens (e.g., 63 different regimens prescribed by 106 providers [14]), and mounting costs and no systems for follow-up (resulting in a doubling of the risk of default [15]).

## Quality Requires Knowledge, Behavior Change, and Enforcement

For well over a decade, the global TB community has promoted public-private mix (PPM) as the response to this challenge (Box 2). At its core, PPM is trying to replace low-quality, inefficient, and potentially expensive health care provision with rapid, affordable, and correct diagnosis and treatment, and monitoring to ensure success.

**Box 2. The Tenets of PPM**

The PPM approach has been structured in several useful ways. The PPM toolkit developed by the World Health Organization and the Stop TB Partnership’s PPM subgroup [16] enumerates the provider types and organizations to engage. These categories are from both the public sector (including government hospitals, social security organizations, prisons, police and military, and academic institutions) and private sector (including nongovernmental organizations [NGOs] and faith-based organizations, corporate health services, private hospitals, private chest physicians and general practitioners, pharmacies, and traditional healers and other unqualified medical practitioners). The tool for conducting a national situational assessment [17] provides further guidance on determining the progress in engaging these groups and discusses related policy issues.

For program design, the initial efforts in PPM have been based on two major concepts. First, the public sector negotiated contracts with private providers to define who does what [18]. Second, to reach individual providers, organizations stepped in to be the intermediaries between public and private sectors. These intermediary organizations—to date, typically NGOs—take on the huge task of aggregating and engaging with individual providers, notifying cases to the national TB programs (NTPs), and coordinating with NTPs for free drugs and follow-up [19].

The quality challenge is related to two issues: insufficient promotion of treatment standards (such as the *International Standards for TB Care* [20]) and limited regulation of those standards. Corrective actions on these two fronts can reinforce each other. In-service education, collaboration, persuasion, and peer support can establish higher standards of health care provision, and regulation can enforce these improved standards to narrow the substantial [9,21] know-do gap between what providers know and what they do in actual practice. Thus, PPM approaches should not choose between education and regulation, since both are necessary [22].

The choice of task mixes—who does what—must be clear and be used to maximize efficiencies [16,23]. Many frontline providers in Asia, and particularly in sub-Saharan Africa, are informal (e.g., traditional healers), resulting in a very low quality of medical care [21,24]. In these circumstances, the best option is to strictly limit the types of activities permitted by these providers, educate only on these few activities (e.g., symptom screening and referral), and enforce the limitations with regulations. The same applies for pharmacists, who should refer, not treat.

Even if qualified doctors are present in the private sector, some countries with minimal TB treatment in the private sector have opted to ban private sector TB drug sales [25]; doctors in these private sectors are expected to refer people with TB to the public sector. Bangladesh presents an interesting middle scenario: although TB treatment was formerly common in the private sector, the free provision of TB drugs by the public sector was widely publicized, leading to a significant, market-driven decrease in the private-sector sales of TB drugs [11,26].

However, in other countries there is such a large private market for TB drugs that a ban is seen as untenable. In this context, regulations focus on two areas. First are efforts to extend laboratory quality assurance systems, previously limited to the public sector, to include private-sector laboratories. Second is to ensure that pharmacies provide TB drugs only with a prescription from a licensed, qualified provider [27,28], in some cases for free [29], and with notification of TB patients by the private sector.

Once the desired change is understood, a key consideration for PPM schemes is their incentive structure. In general, incentives for TB PPM have been based on a combination of factors: motivation and moral persuasion; free extras (training, performance recognition, and free diagnosis and treatment); and, in some cases, limited financial compensation. With any financial mechanism comes major challenges for record verification and then timely and transparent disbursement. Thus, financial incentives make the most sense when patient volumes are large (resulting in administrative economies of scale), such as in laboratories or hospitals with chest physicians, and when the medical or economic stakes are highest, as in the management of multidrug-resistant TB.

Ideally, PPM activities establish a new norm in behavior that persists after the intervention is withdrawn. But results based only on motivation alone may be unstable, and, anecdotally, success can be dependent on charismatic local leadership [30]. This may explain the success of pilots but the subsequently variable results in country-wide implementation.

PPM is really a form of behavior change for both sides: for the public sector in their approach to engaging the private sector and for the private sector in changing their practices. It requires all the usual lessons from this field, such as the use of repetition, learning from the actions of others (social cognitive theory), and peer and group interventions [31]. One of the potential groups for implementing this behavior change is the professional association or society. Members of these associations are well situated to provide behavior reinforcement messages to their peers [32]. However, most associations have not achieved the organizational scale required for wide coverage of the education function, and they rarely have the manpower for on-site inspections or the mandate to enforce regulations.

The difficultly with regulation is, indeed, enforcement. This mandate would typically fall to government, but professional licensing schemes are often cursory, with no quality component. Does the public sector accept—and have the capacity to address—its responsibility not only to run public facilities but also to regulate and oversee private facilities? India and Indonesia are moving swiftly in this direction (including accreditation for hospitals and laboratories and more formal licensing requirements for providers), and there are signs that others will do so as economies grow. In Cambodia, for example, certain provinces are exploring if the regular renewal of private pharmacy and clinic licenses by the public sector can be used as an opportunity to enforce certain quality and regulation issues [30].

## Consolidation of Supply Makes Everything Easier

All of this training and regulation can be managed if there are enough personnel to reach all providers. This engagement task is more manageable when providers are already well connected to the broader health system, and can be reached as a group. For example, some of the quick wins in the PPM world have come from hospital and medical college engagement [33]. Hospitals constitute a substantial unit for engagement, with large patient flows that include many people with TB symptoms. A directly observed treatment short-course (DOTS) clinic in a hospital can do TB reporting and defaulter tracing for the entire hospital—and indeed, for surrounding private providers [34]—and the hospital administration can disseminate new policies and practices to all staff. This has allowed hospitals to contribute up to 10% and 50% of case detection in parts of the Philippines [35] and Indonesia [36], respectively.

Outside of such settings, the numbers become more challenging. India has over 750,000 chemists and pharmacists [37]; most see only a handful of people with TB symptoms a week. For a traditional PPM scheme, each one of these providers needs to be visited individually by either the public-sector TB worker or the intermediary organization.

The PPM intermediary organization (Box 1) is, however, the beginning of an organizing or consolidating force in an otherwise fragmented health care system made up of individual private providers. The intermediary organization networks providers to take part in education, reporting, and regular supervisory visits. Tactics have varied: Bangladesh schemes focus on reaching as many providers as possible, but with a relatively informal (verbal) referral system [26], whereas schemes in Cambodia use triplicate referral forms [30].

Intermediary organizations can benefit from performance-based financing, in which payment is not for inputs but for outputs such as case notifications or cures. Although misaligned incentives are possible, in general this encourages optimization. An example is India’s urban pilot of private-provider interface agencies (PPIAs), which is using a basket of services for privately treated patients, such as vouchers for free TB tests and drugs. The PPIAs make maximal use of information technology such as mobile phones, call centers, electronic notifications and drug vouchers, and linkages with universal ID numbers or large databases for social security. These mechanisms make it possible to educate and maintain contact with the large number of providers, notify cases to the NTP, and help with patient follow-up [38]. In PPIAs, private providers can retain their patients and even offer several value-added services [39].

Consolidation of private laboratories by an intermediary agency is also feasible. The Initiative for Promoting Affordable and Quality Tests (IPAQT) in India provides over 100 accredited laboratories with concessionary pricing in exchange for their case notification to the NTP and passing on the price reductions to patients for WHO-endorsed TB tests [40,41].

These various types of PPM schemes can clearly boost TB case detection and treatment outcomes. In 2013, PPM with private-sector providers alone yielded 14% of all TB notifications in Ethiopia, Nigeria, and Pakistan, 15% in Tanzania, 20% in Myanmar, and 21% in Kenya [42]. Intermediaries were most often NGOs but also included professional associations (e.g., in Myanmar and Indonesia) and social franchise organizations (in Myanmar and Pakistan).

However, as economies grow and TB incidence drops, it will be more and more difficult to sustain interest and funding for such TB-only schemes. We need to look for new ways to promote the connection of individual health care providers to each other and to networks and systems in the broader health system, and for opportunities to include TB in any cross-cutting initiatives on health care consolidation, rationalization, and organization [43].

## Economic Growth and More Sustainable Opportunities

If it is the atomization of the health care system that is PPM’s biggest challenge, then economic growth is bringing some potential solutions. Since 1950, 13 economies have grown at an average of 7% or more for 25 years or longer [44], and many other countries are on an earlier but similar trajectory. The resulting changes in health financing [45] will bring changes in health systems overall and in the opportunities for engaging private providers.

Social franchises [46,47] or social businesses [34,41] provide one possible structure for this engagement. These schemes still fall under the category of intermediary organizations, as noted above. However, they combine their programmatic goals in public health with business structures and aim to move, ultimately, beyond a project status and into the territory of a self-sustaining business. Rather than focusing only on what the public sector “needs” and what the private sector “should” do, these organizations aim for a logic that works for the private entity itself. Integration of multiple disease areas is a critical characteristic of these initiatives [43]: Non-TB indications may provide greater patient volumes and prove a greater draw for providers [48], but TB is included as a requisite part of the business model.

Income from these businesses could in theory lead to sustainability, but there are two major concerns. First, the amount of this income is currently far from sufficient to cover the total operating expenses [43,49]. Presumably, financial flows may increase as economies develop. But second, the income is almost entirely from out-of-pocket expenditures by patients—a problem for low-income communities in which TB is most prevalent. In the future, social franchises and businesses could make ideal recipients for results-based financing from governments and for payments from growing national health insurance (NHI) schemes.

Results-based financing has been tried both by funding agencies and by governments for disbursement of their own money, but governments can be reluctant to directly fund private-sector entities with domestic resources. Thus, there has been limited work on creating efficient, results-based pathways from governments to private providers.

By contrast, many countries have growing NHI schemes aimed at both public and private providers; these are an integral component of their push towards universal health coverage (UHC) [50,51] and will be a critical factor in future engagement of private providers.

A number of NHI schemes have reimbursements for TB, including for TB treatment by private-sector physicians [43,52]. The schemes may be a two-edged sword [53]: public TB programs may struggle to secure alternative financing to maintain certain essential public health functions [54] even as the private sector finds itself with a more stable, self-sustaining income stream for private-sector TB treatment. In addition, NHI schemes often start by covering people with formal employment, who are typically richer and thus at a lower risk for TB [51].

Many of the teething problems with these schemes relate to the extra administrative burden, which may bring enrollments or reimbursements to a halt, thus removing the incentive to participate in the insurance scheme [52]. However, it is this very administrative effort (combined with information technologies) that is linking together providers and thus providing the structure and opportunity for PPM. These are promising experiments worth watching.

Can the processes inherent in health insurance schemes address the TB quality issue? Theoretically, NHI schemes could include payments to private providers for certain desirable objectives, such as TB treatment completion. However, the field lacks the microeconomic analysis of providers to help guide the design of such incentives [43]. In addition, most TB treatment occurs in a primary health care setting in which providers are paid based on caseload (capitation) rather than specific procedures. This makes it difficult to tie payment to specific diagnostic and treatment behaviors [53].

However, several countries are experimenting with an interesting combination of regulation and insurance [53]. For example, enrollment of providers in the NHI scheme, and continuing reimbursements, can be made contingent on achieving certain accreditation or licensing requirements, including laboratory standards, further education on TB standards, or prompt reporting of TB cases [20,55]. The latter requirement also needs an efficient (meaning, usually, electronic [56,57]) system for mandatory notification.

With economic growth, the low-quality, informal private medical sector generally tends to diminish, and health services become more organized in other ways. The establishment of large private pharmacy chains in the Philippines [58] and lab networks in India are examples of this trend; market-based interventions can encourage this process [59]. As with the hospital example above, this reduces the number of distinct entities that need to be engaged by TB organizations.

## Evolution of Health Workforces

PPM highlights some difficult choices that need to be tackled in developing health systems. One of these issues is dual practice. In many low-income settings in Asia, the public-sector salaries for health care workers are insufficient to sustain a family. Therefore, the doctor or pharmacist practices in the public sector for a few hours, and the remaining time is spent in private practice. Such a pattern holds for an estimated 90% of doctors in some of these countries [26,30].

Such a system certainly constitutes an unhealthy linkage between public and private, as it sets up some unfortunate dynamics, and dual standards of care. Public-sector doctors have an incentive to provide substandard service or to neglect certain essential public-sector services, such as X-rays, so that their private practice will get more referrals. In addition, when the doctor or pharmacist is working in the public sector, their private business often stays open. During these times, in the absence of the qualified provider, drugs and advice are typically dispensed by unqualified relatives or employees [30]. Broader solutions, such as realistic, performance-based compensation in the public sector and regulation of dual practice [60], will be an integral part of PPM solutions.

## Conclusion: Towards a More Systemic and Scalable PPM

PPM is moving from pilots to widespread implementation and is contributing to a larger percentage of TB notification and treatment. But scale, sustainability, and affordability remain a major concern. In the meantime, new opportunities have arisen in the form of social businesses, national health insurance schemes, payment reform, regulatory regimes, information technologies, and consolidation of other health care structures. All these efforts in TB are highlighting the complex policy choices that lie ahead and are providing the motivation to tackle initiatives that will benefit the entire health sector.

## Abbreviations:

DOTS: directly observed treatment short course
IPAQT: Initiative for Promoting Affordable and Quality Tests
NGO: nongovernmental organization
NHI: national health insurance
NTP: national TB program
PPIA: private-provider interface agency
PPM: public-private mix
TB: tuberculosis
UHC: universal health coverage
